# Embryonic Development of Heart in Indian Buffalo (*Bubalus bubalis*)

**DOI:** 10.1155/2014/293675

**Published:** 2014-09-30

**Authors:** Anuradha Gupta, Neelam Bansal, Varinder Uppal

**Affiliations:** Department of Veterinary Anatomy, Guru Angad Dev Veterinary and Animal Sciences University, Ludhiana, Punjab, India

## Abstract

The present study was conducted on 35 buffalo foetuses from 0.9 cm CVRL (32 days) to 99.5 cm CVRL (298 days) to observe the morphogenesis and histogenesis of heart. The study revealed that, in 0.9 cm CVRL buffalo foetus, heart was unseptated and tubular which was clearly divided into common atrial chamber dorsally, primitive ventricles ventrally, primitive outflow tract with bulbous cordis region proximally, and aortic sac distally at 1.2 cm CVRL. Septum primum appeared at 1.9 cm CVRL whereas the truncal swellings and fold of interventricular septum appeared at 2.5 cm CVRL foetus. At 3.0 cm CVRL septum primum, endocardial cushions, septum secundum, and foramen ovale were observed. At 7.6 cm CVRL the endocardial cushions fused to form right and left atrioventricular openings and ventricular apex became pointed. Interventricular canal was obliterated and four-chambered heart was recognised along with atrioventricular valve, chordae tendineae, and papillary muscles in 8.7 cm CVRL (66 days) buffalo foetus. The endocardium as well as epicardium of the atria was thicker as compared to ventricle, whereas the myocardium of atria was thin as compared to ventricles in all the age groups. All the internal structures of heart were well differentiated from 50 cm CVRL onwards. The detailed structural components of buffalo heart during prenatal period have been discussed in the present paper.

## 1. Introduction

Buffalo (*Bubalus bubalis*) is widely spread over whole of Asia and has been an integral part of livestock economy for over 5000 years providing draft power, milk, meat, and hides [1]. In India, buffaloes are preferred over cattle because of high milk fat content which fetches higher market price and also for their ability to utilize coarse feeds better than cattle [2]. Unfortunately, this species of animals did not receive the attention of researchers in accordance with its merits, which resulted in decline of buffalo population [3]. One of the most important scientific goals in this century is integration of basic research with its clinical significance. The recent advances in elucidating myocardial structure and function have made a paradigm shift in research and provided a promising ground for new integrative knowledge of heart structure and function [4]. As heart is the first organ to form and function during development [5], it undergoes marked structural remodelling during development. Therefore, the study of cardiac myocytes, differentiation, development, and maturation in buffalo is very important to know the mechanism of fundamental development, congenital malformation, and inability of mature heart to regenerate.

In the available literature, the information on the embryonic development of heart in chick [6], pig [7], and human [8] has been reported, but the studies related to development of foetal heart in buffalo are still lacking. So, the present research work was undertaken to correlate the function of heart in relation to its structure in different age groups and as a diagnostic tool in congenital abnormalities during foetal life.

## 2. Materials and Methods

### 2.1. Collection of Samples

The present study was conducted on heart samples of 35 buffalo foetuses of different gestational age which were obtained from pregnant nondescript buffaloes slaughtered at Abattoir, Saharanpur, and presented at Veterinary Clinics, GADVASU, Ludhiana (Table 1). The foetal body length was measured as curved line in centimetre with the help of inelastic thread along the vertebral column between the most anterior parts of frontal bone to the rump at ischiatic tuberosity and designated as curved crown rump length [9]. The approximate age of the foetuses was calculated by using the formula given by [10].

(1) $$\begin{array}{c} Y=28.66+4.496X\;\left(\text{CVRL}<20\;\text{cm}\right)\\ Y=73.544+2.256X\;\left(\text{CVRL}\geq 20\;\text{cm}\right), \end{array}$$

where *Y* is age in days and *X* is CVRL in centimeters.

Based on CVRL the foetuses were divided into three groups.

*Group I*. Foetuses of CVRL between 0 and 20 cm.

*Group II*. Foetuses of CVRL between >20 and 40 cm.

*Group III*. Foetuses of CVRL above 40 cm.

### 2.2. Fixation and Processing

In small sized foetuses up to 3.2 cm CVRL, the whole mount was taken as it is difficult to collect heart at this age. In the foetuses from 3.2 to 19 cm CVRL, complete heart was taken out and serial sectioning was done on the foetuses/heart samples. From 19.5 to 99.5 cm CVRL buffalo foetuses, the small tissue samples were collected from different components of heart and were fixed in 10% neutral buffered formalin (NBF) and Bouin's fixatives. Once the fixation was achieved, the tissues were processed for paraffin block preparation by acetone-benzene schedule [11]. The blocks were prepared and sections of 5–7 *μ*m thickness were cut and obtained on clean glass slides with rotary microtome. The paraffin sections were stained with haematoxylin and eosin for routine morphology, Masson's Trichrome for connective tissue, Verhoeff's stain for elastic fibers, and Gridley's stain for reticular fibers.

## 3. Results

### 3.1. Morphogenesis

#### 3.1.1. Formation of Heart Tube

**Figure 1:**
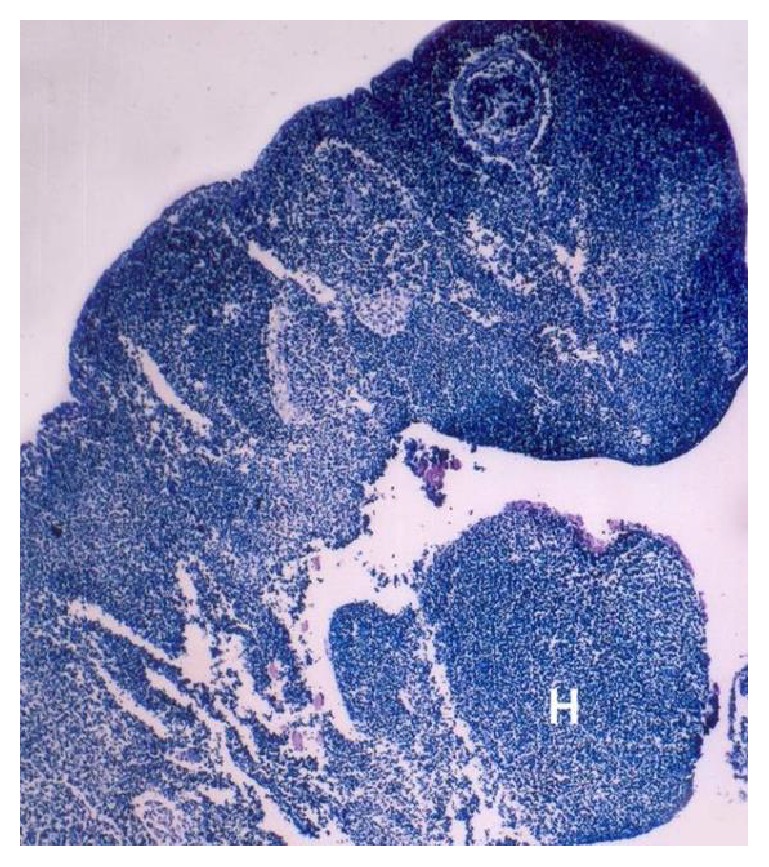
0.9 cm CVRL foetus showing cardiac prominence (H). Hematoxylin and eosin ×20.

At 0.9 cm CVRL, the buffalo foetal heart was unseptated and tubular just ventral to the foregut. The heart was triangular in shape with a broad base and narrow apex (Figure 1). The base has indentations due to presence of mandibular arch and hyoid arch.

#### 3.1.2. Cardiac Loop Formation

**Figure 2:**
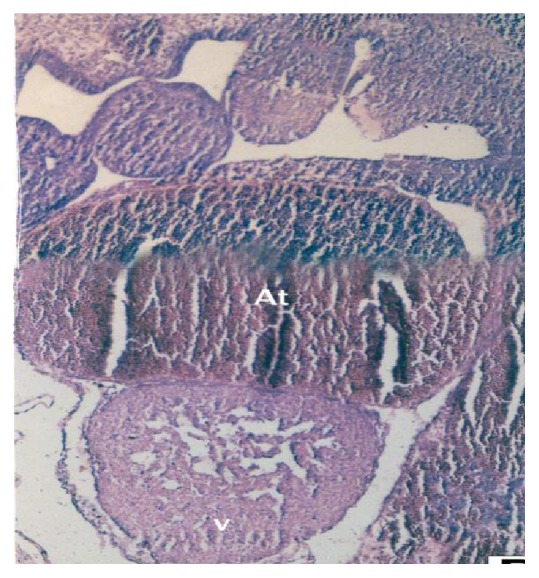
1.2 cm CVRL foetus with mesenchymal cells in atrium (At) and young myocyte cells in ventricle (V). Hematoxylin and eosin ×100.

The buffalo foetal heart of 1.2 cm CVRL was clearly divided into common atrial chambers dorsally, primitive ventricle ventrally, primitive outflow tract with bulbous cordis region proximally, and aortic sac distally. Between the atrium and ventricle the heart remained undilated and this narrow region was the atrioventricular canal (Figure 2). The most cephalic part of cardiac tube persisted as truncus arteriosus, which connected the ventricle with the ventral aortic root in foetal heart of 1.2 cm CVRL. The transitional region where the ventricle narrowed down to join the truncus arteriosus was the conus.

#### 3.1.3. Partitioning of Heart

**Figure 3:**
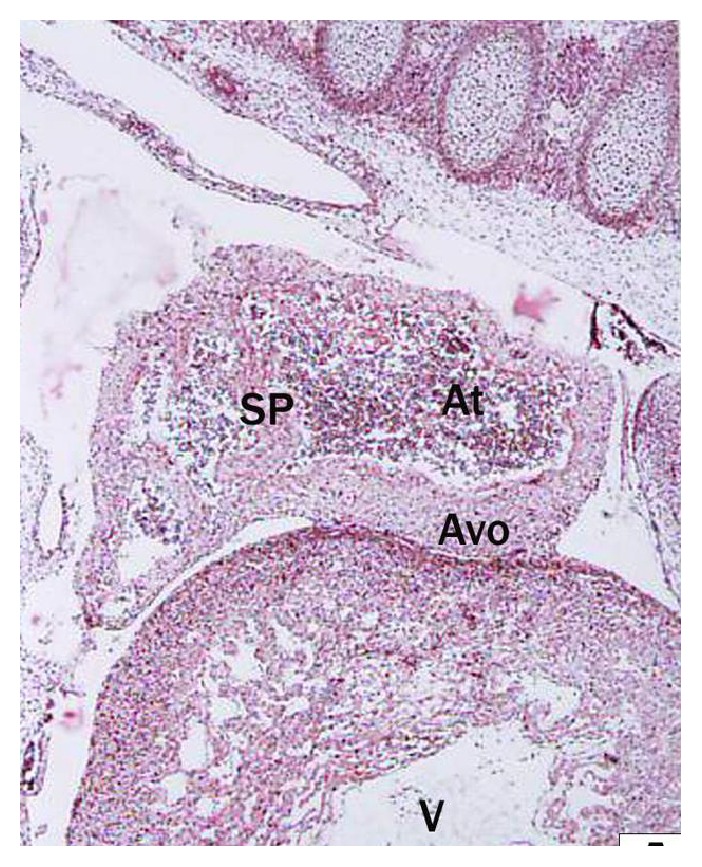
Longitudinal section of buffalo foetal heart of 3 cm CVRL showing atrium (At) and ventricle (V). Hematoxylin and eosin ×40.

In 3.0 cm CVRL foetal heart, the two conspicuous masses of loosely organized mesenchymal tissue called endocardial cushions developed (Figure 3) in dorsal and ventral wall of atrioventricular canal. In 7.6 cm CVRL foetus the dorsal and ventral endocardial cushions met and occluded the central part of the canal and separated it into right and left atrioventricular canals.

**Figure 4:**
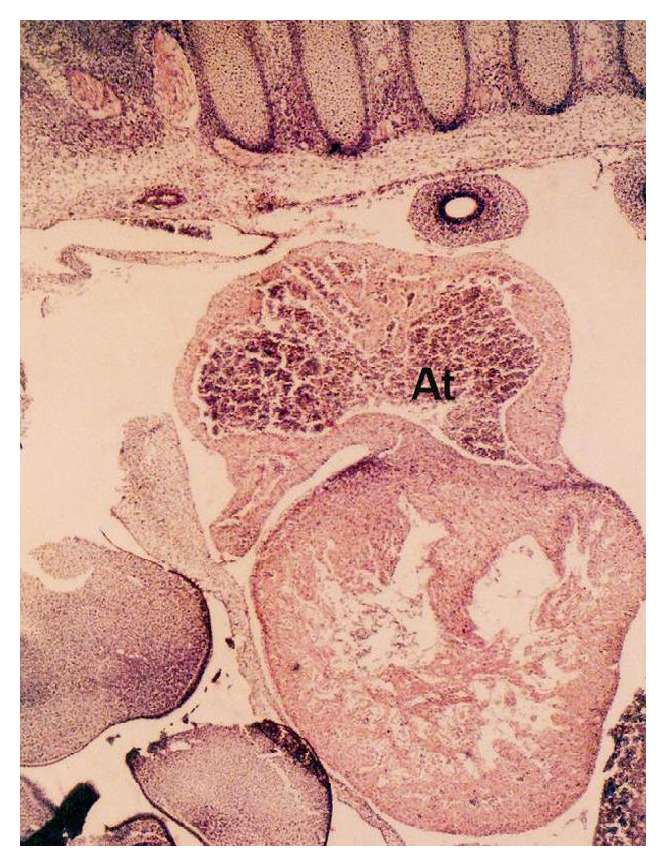
Buffalo foetal heart of 1.9 cm CVRL showing septum primum (SP) in atrium (At), ventricle (V), and atrioventricular opening (AVO). Hematoxylin and eosin ×100.

*(i) Partitioning of Atrium*. At 1.2 cm CVRL, atrium was composed of mesenchymal tissue. The division of common atrium into right and left chambers was first achieved by appearance of crescent shaped septum primum in 1.9 cm CVRL buffalo foetus. It was composed of a thin layer of young cardiac muscles covered by thin endothelium (Figure 4). The septum primum projected ventrally into the lumen towards endocardial cushions. Ostium primum located between free margins of septum primum and endocardial cushions was reported at 3.0 cm CVRL. The later was gradually occluded by the further enlargement of cushions but before complete closure, the number of perforations appeared within the septum which coalesced to form ostium secundum. The septum secundum which appeared right to the septum primum completely divided the atria into right and left parts. The foramen ovale was first time noticed at 3.0 cm CVRL between septum primum and septum secundum. The size of foramen ovale did not change with the gestational age and therefore the proportion of the foramen ovale to inferior vena-cava decreased with gestational age.

*(ii) Partitioning of Ventricle*. In the buffalo foetus of 3.0 cm CVRL, the ventricle was partitioned by the interventricular septum extended upward from the ventral side of heart toward the endocardial cushions leaving an opening in the interventricular foramen (Figure 3). It was made up of majority of young myocytes along with mesenchymal cells.

**Figure 5:**
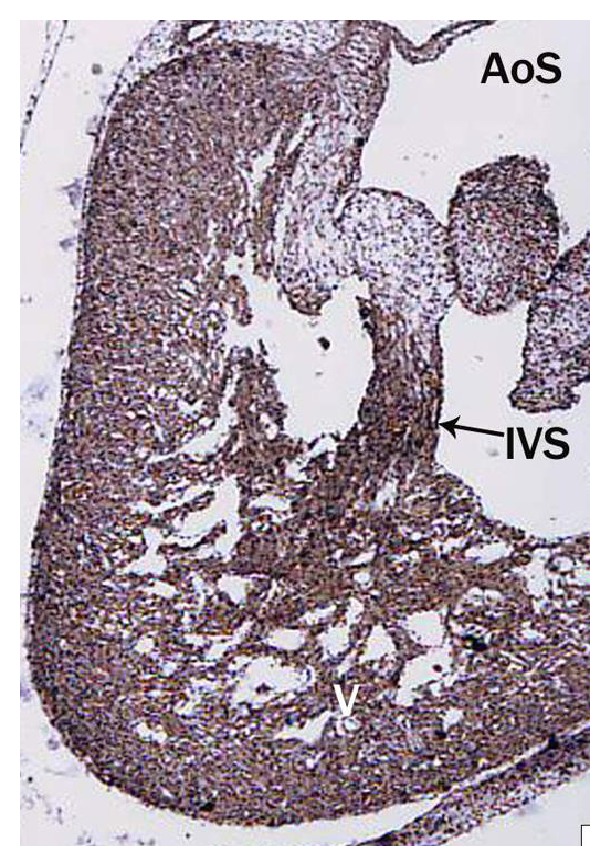
Buffalo foetal heart of 2.5 cm CVRL showing truncal ridges of aortic sac (AoS) and interventricular septum (IVS). Hematoxylin and eosin ×100.

*(iii) Partitioning of Truncus Arteriosus*. The buffalo foetus of 2.5 cm CVRL showed the presence of truncal swellings in the lumen of truncus arteriosus. These ridges were spiral and fused to form spiral septum and divided the truncus into aortic and pulmonary trunks (Figure 5). The aortic and pulmonary valves also developed from these ridges observed in 3.2 cm CVRL foetus.

*(iv) Closer of Interventricular Foramen*. In 8.7 cm CVRL buffalo foetus the final closure of the interventricular foramen was possible by masses of endocardial cushions tissue from three sources—interventricular septum, endocardial cushions, and truncoconal ridges. By the time the interventricular septum was completed the right ventricle continued into pulmonary trunk and left in aorta.

### 3.2. Histogenesis

In the buffalo foetus of 0.9 cm CVRL the initial heart tube was composed of mesenchymal cells lined by mesothelium (Figure 1). At 1.2 cm CVRL buffalo foetus, the heart tube was comprised of two layers namely; endocardium and epimyocardium (Figure 2). Epimyocardium was further differentiated into myocardium and epicardium in 3.2 cm CVRL foetal heart.

#### 3.2.1. Endocardium

**Figure 6:**
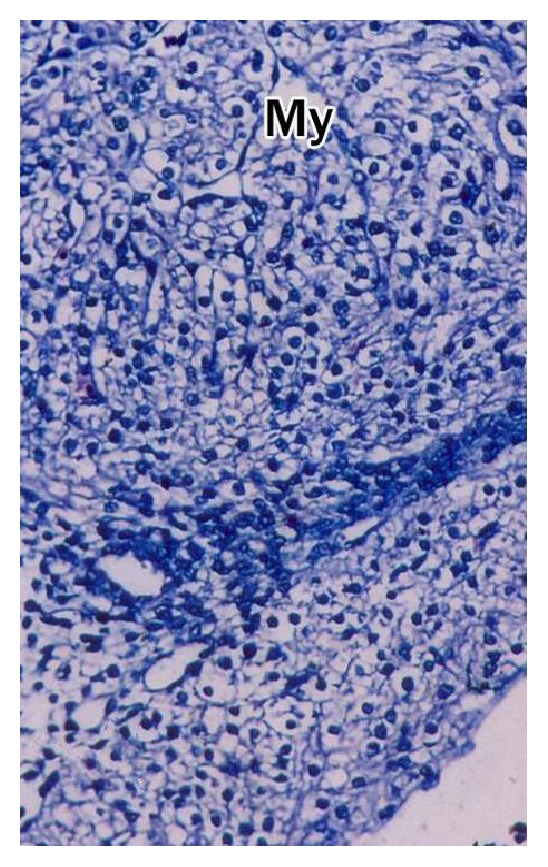
Ventricle of 3.2 cm CVRL buffalo foetal heart showing differentiating myocytes (My) and pericardium (P). Hematoxylin and eosin ×100.

**Figure 7:**
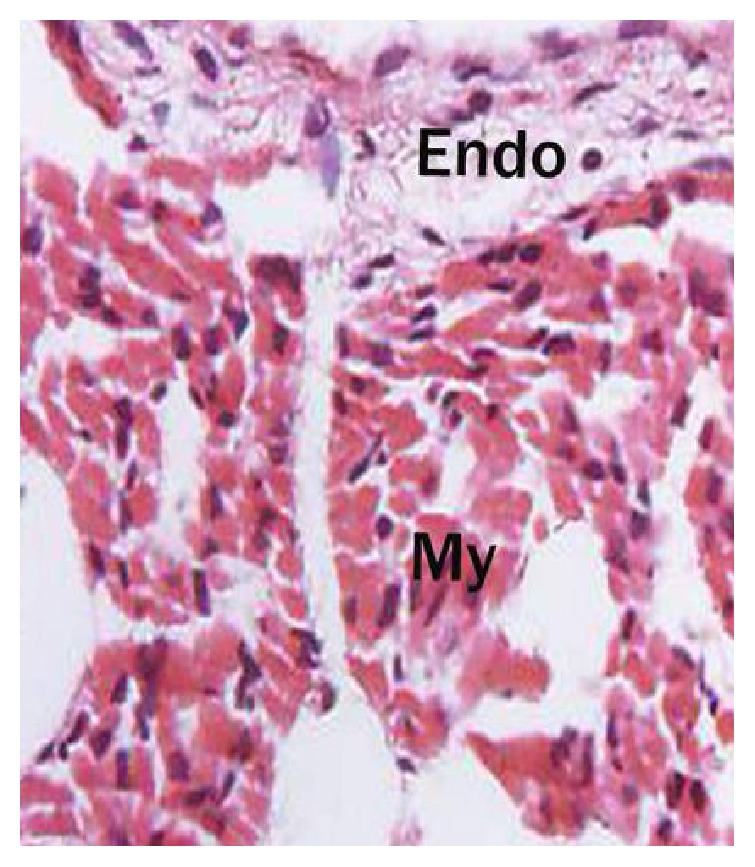
Section of ventricle of 50 cm CVRL buffalo foetal heart showing two layers of endocardium (Endo) and myocardium (My). Hematoxylin and eosin ×400.

It originated as a single layer of flattened endothelium in the ventricle of 1.2 cm CVRL foetus, whereas it was differentiated both in the atrium and in ventricle at 1.9 cm CVRL (Figure 4). A lightly stained cellular matrix, that is, cardiac jelly, was also observed below the endocardium in 3.2 CVRL foetus (Figure 6). The endothelium was found to be continued with tunica intima of pulmonary artery and aorta and endothelium of semilunar and cardiac valves in 11 cm CVRL foetus. The increased size of endocardial cells was observed at 3.2 cm CVRL foetus. The elongation of endocardial cells and presence of mitosis suggested that endocardium grew out due to increase in cell number and by redistribution of preexisting cells. The endothelial cells were elliptical to rounded shape with central nucleus and bulging towards the lumen. In 13 cm CVRL foetus the thin layer of subendothelium was observed with large number of differentiating Purkinje fibres. It was composed of loose connective tissue along with few elastic and reticular fibers, which were arranged parallel to the endocardium. In group II, the thickness of this layer has increased with the formation of more fibrous tissue and blood vessels. At 50 cm CVRL the subendothelium was further differentiated into inner (dark) and outer (light) subendothelium (Figure 7). Inner subendothelium was composed of dense irregular connective tissue with collagen fibres, elastic fibers, and few smooth muscle cells. The outer one was in direct contact with myocardium and was composed of irregularly arranged connective tissue that merge with collagen and elastic fibres surrounding the adjacent cardiac muscle along with Purkinje fibres, blood vessels, and lymphatics.

#### 3.2.2. Myocardium

**Figure 8:**
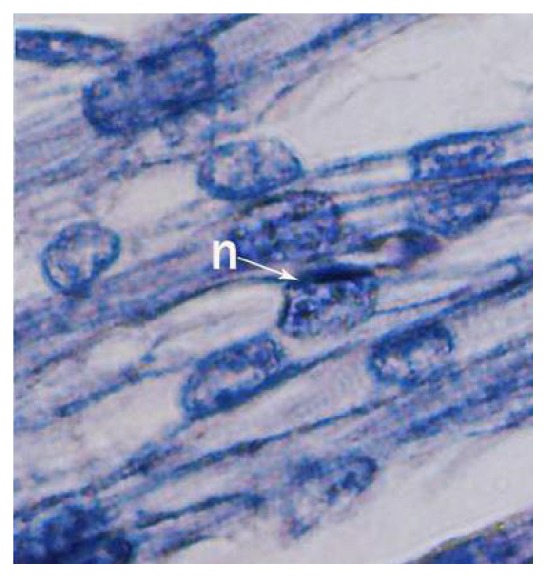
Section of 99.5 cm CVRL buffalo foetus showing cardiac muscle striations. Phosphotungstic acid hematoxylin ×1000.

**Figure 9:**
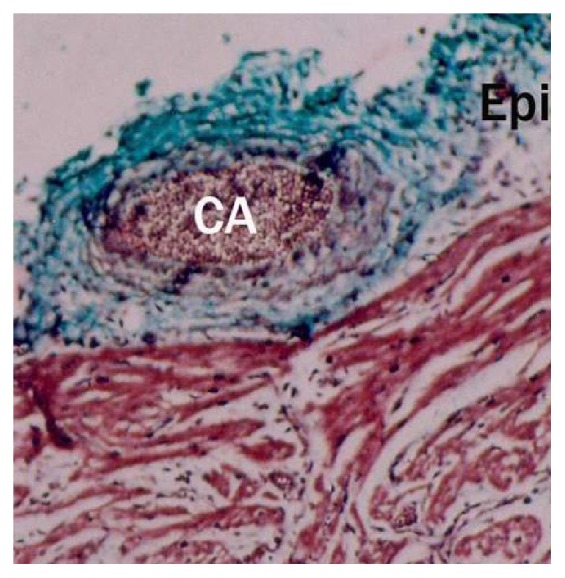
Buffalo foetal heart of 13 cm CVRL showing more collagen fibers in epicardium (Epi) than myocardium (My). Masson's trichrome ×100.

The buffalo foetus from 1.2 cm to 3.0 cm CVRL showed the presence of trabeculae in the ventricles which was well-differentiated at 3.2 cm CVRL. Towards the epicardium, the myocytes formed a dense layer which was highly trabeculated in the lumen side (Figure 8). At 3.2 cm CVRL, the spaces among the cardiac myocytes were invaded by mesenchymal cells that would differentiate into intracardial connective tissue. The intercalated discs began to form at the end-to-end junctions of myocytes in groups I and II which were well appreciable in group III (Figure 8). The trabeculations migrated towards the epicardium in 4.2 cm and 4.8 cm CVRL foetuses. In 7.0 cm CVRL, the trabeculation reached up to epicardium with development of large intercellular spaces and decreased cardiac jelly. The myofibers were sparse in 7.6 cm and 8.7 cm CVRL buffalo foetuses due to degeneration of myocytes. At 11 cm CVRL, the trabeculae decreased in height with less intercellular spaces. The myocardium of buffalo foetal heart at 13 cm CVRL showed remarkable changes. The musculature of left ventricle was outer, middle, and inner layers which were oriented in longitudinal, circular, and oblique directions, respectively (Figure 9). In the right ventricle, myocardium was comparatively thin and had two groups of fibers, that is, inner and outer layers. At 18.8 cm CVRL, bundles of cardiac muscle cells were observed which were encircled by a layer of loose connective tissue, that is, perimysium, and the individual muscle fibers was surrounded by a thin layer of connective tissue as endomysium in group III (Figure 7). The cellular and fibrous components of endomysium and perimysium were increased with advancing age of foetus.

The myocardium of atria was thin as compared to ventricles and was loosely arranged in various directions, namely, circular, longitudinal, and oblique. The connective tissue fibers composed of collagen and elastic fibers were more in atria (Figure 14). Myocytes were observed as spindle shaped mononuclear cells with large number of longitudinally oriented myofibrils. The striations of muscle fibers were observed in all the age groups. The cardiac muscles were branched and anastomosed and end-to-end junctions of the muscle cells, that is, intercalated disc, were noticed. The nucleus of myocyte was single, large oval, or spherical with one or two nucleoli (Figure 8). Proliferation of myocytes and transition of the mononucleated myocytes to binucleated was observed. The myocytes in right ventricle of foetal heart were larger and contained more myofibrillar material than left ventricle. The atrial myocytes were smaller than ventricular myocytes. In between the myocytes fibroblasts, mesenchymal cells and few lymphocytes were seen.

#### 3.2.3. Epicardium

At 0.9 cm CVRL, the foetal heart was lined by the mesothelial cells of epicardium which was observed as epimyocardium from 1.2 cm to 3.0 cm CVRL buffalo foetuses. The latter was differentiated into a thick myocardium and a thin epicardium at 3.2 cm CVRL (Figure 6). The epicardium was composed of single layer of squamous cell in atria and ventricles from 3.2 cm to 4.8 cm CVRL foetuses. At 7.0 cm CVRL a noticeable gap was found between the epicardium and myocardium as subepicardium which was composed of loose connective tissue containing blood vessels. In 8.7 cm CVRL foetus the subepicardial space became enlarged which showed subepicardial thickenings at places. The continuity of epicardium with parietal pericardium was noticed in 11 cm CVRL foetus where the pulmonary artery was leaving the heart. At 13 cm CVRL, the epithelial cells lied on the thick stroma of connective tissue composed of enlarged collagen fibres, few elastic and reticular fibres, arteries, veins, and lymphatics (Figure 9). In 27 cm CVRL foetus deeper part of epicardium contained large nerve bundles, smooth muscle cells, ganglionated and nonganglionated nerve fibres, and adipose tissue. The abundance of collagen fibres was seen at 87.5 cm CVRL (Figure 11). Some of the fibrous tissue from epicardium migrated towards endocardium through the myocardium in group III. The subepicardial layer of ventricular wall of buffalo foetus of 99.5 cm CVRL was very thick and consisted of large collagen fibres, elastic fibers, and loose areolar tissue with few smooth muscle cells. Epicardium of ventricles was continued with aorta and pulmonary arteries as tunica adventitia.

#### 3.2.4. Moderator Band

At 19 cm CVRL, it was composed of Purkinje fibers, thin layer of myocardium, and endocardium. The ventricular endocardium continued as the moderator band endocardium and became thick as compared to ventricular endocardium (Figure 12). In 25 cm CVRL foetus, an increase in the myocardial layer thickness and subendothelial space was also noticed. In the 99.5 cm CVRL foetus two layers of myocardium (outer longitudinal and inner oblique) were observed along with blood vessels and nerves in the subendocardium. The Purkinje network in the subendocardial was elliptically arranged in the ventricles and was surrounded by the fibrous sheath along with connective tissue fibres that separated the specialized myocytes from the ordinary myocytes which help in conducting fast stimuli without any disturbance. These Purkinje fibers enclosed in capsule in the right moderator band were forming the right bundle branch, which ran from the bundle through interventricular septum and continued through moderator band to the anterior papillary muscles. The Purkinje fibers constituted about half of the thickness of the moderator band in groups II and III. Large blood vessels and nerve fibers were also noticed in moderator band. The artery of moderator band made anastomotic connections with right coronary artery. Purkinje fibers were broad and irregular and contained less myofibrils than ordinary myocytes (Figure 16). In cross section, the nucleus was round and the chromatin of the nucleus was more dense and compact than the nucleus of ordinary myocardial cells.

#### 3.2.5. Atrioventricular and Semilunar Valves

**Figure 10:**
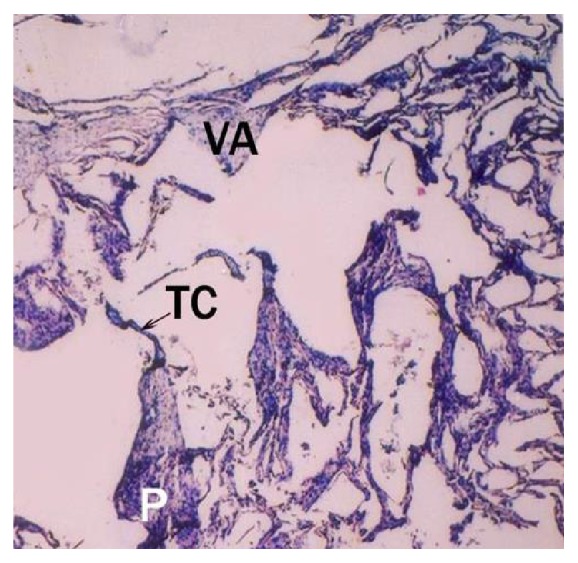
8.7 cm CVRL buffalo foetus showing tricuspid valve (VA), chordae tendineae (TC) and papillary muscles (P) in ventricle (V). Hematoxylin and eosin ×40.

**Figure 11:**
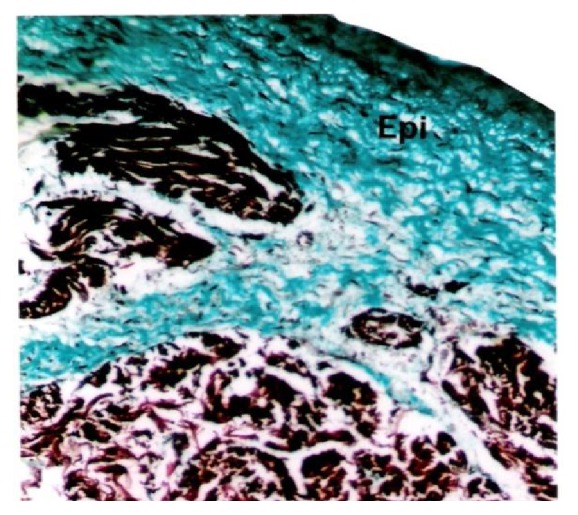
Buffalo foetal heart of 87.5 cm CVRL showing collagen fibers in epicardium (Epi) and myocardium (My). Masson's trichrome ×200.

**Figure 12:**
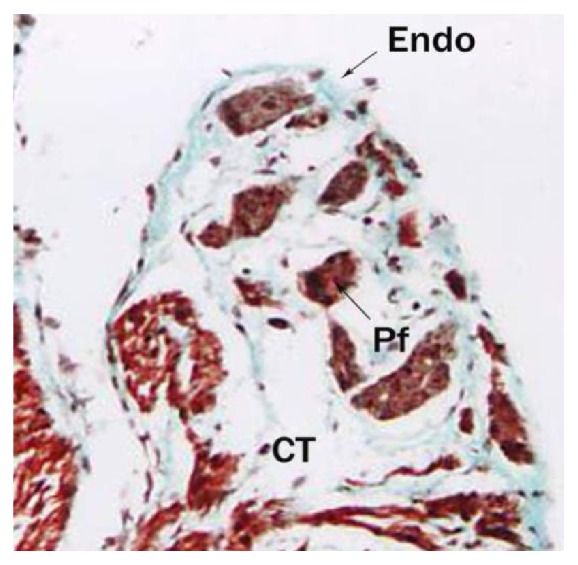
Buffalo foetal heart of 19 cm CVRL showing the Purkinje fibers (Pf), connective tissue (CT), and endocardium (Endo) in right bundle branch. Masson's trichrome ×100.

At 8.7 cm CVRL, atrioventricular valves were first marked at the point where atrioventricular canal opened into ventricles. These valves projected into ventricles as triangular flaps and were lined by the flattened endothelial cells (Figure 10). These consisted of large number of mesenchymal cells with fibroblasts, collagen fibers, and elastic fibers in first group which became cellular with age. Few elastic fibres, numerous blood vessels, and spindle shaped cells were identified in subendothelial zone particularly at atrial surface of the leaflets in 11 cm CVRL.

**Figure 13:**
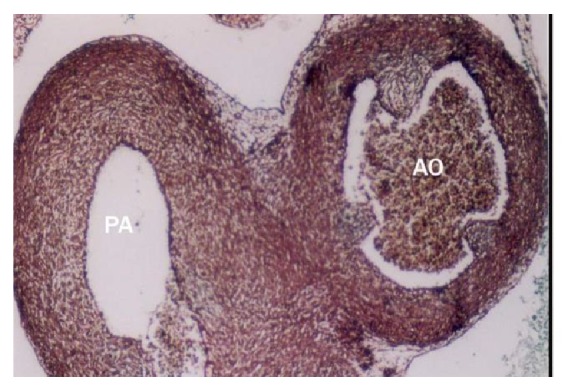
Pulmonary artery (PA) and aorta (AO) of 4.8 cm CVRL showing semilunar valve in aorta (AO). Masson's trichrome ×200.

**Figure 14:**
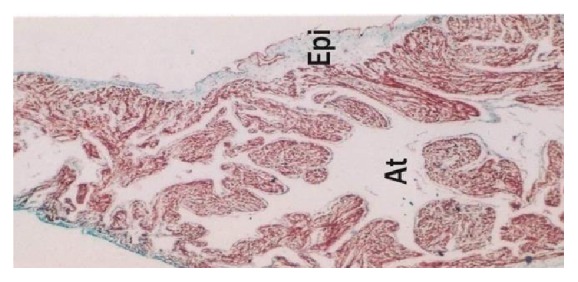
Buffalo foetal heart of 10.5 cm CVRL showing collagen fibers in epicardium (Epi) of atrium (At). Masson's trichrome stain ×40.

**Figure 15:**
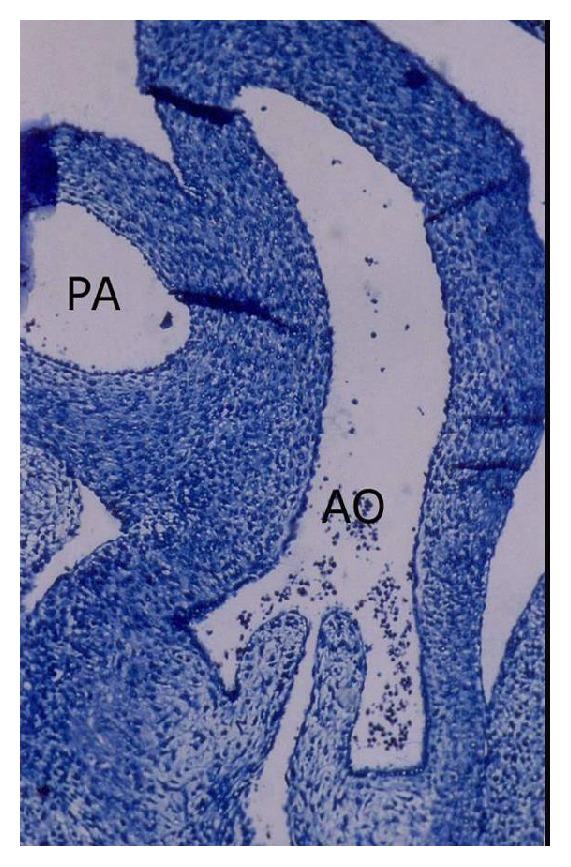
Buffalo foetal heart of 2.5 cm CVRL showing semilunar valve of aorta (AO) and pulmonary artery. Hematoxylin and eosin ×100.

**Figure 16:**
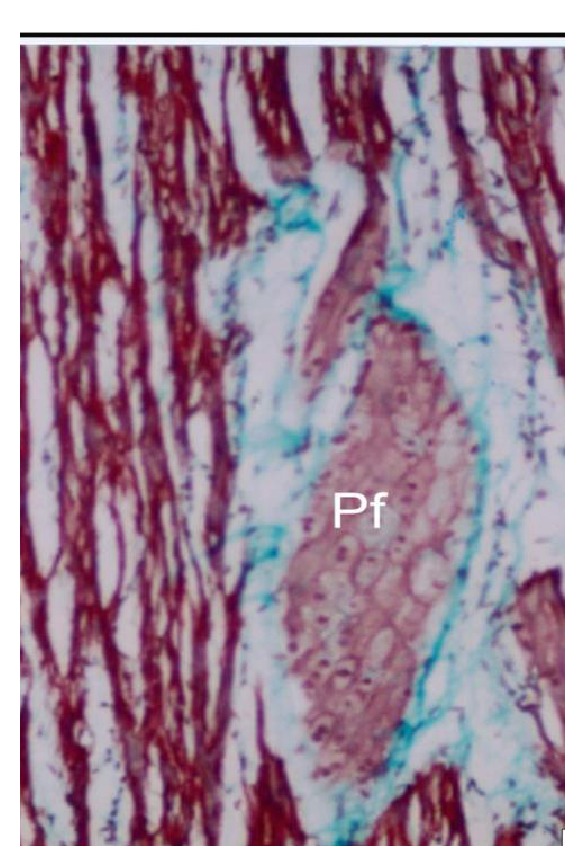
Buffalo foetal heart of 99.5 cm CVRL (298 days) showing Purkinje fibers (Pf) in interventricular septum. Masson's trichrome stain ×200.

The earliest anlage of the semilunar valves was first observed in 2.5 cm CVRL buffalo foetus in aorta (Figure 15) and pulmonary artery. These cusps were composed of a core of loose mesenchymal tissue covered by the flattened endocardium. In 3.2 cm CVRL, the endothelial lining towards ventricular surface was smooth and towards atrial surface was irregular. From 3.2 to 4.8 cm CVRL, the excavation in the atrial face of the cusps resulted in the achievement of their final shape (Figure 13). The fibroblasts and collagen fibers were noticed in the subendothelium at 11 cm onwards. The myocardial cells of outflow tract invaded the valvular tissue and formed the prominent fibrous layer. In groups II and III the fibroblasts were decreased and collagen fibers were increased. It was found that the semilunar valves were poorly vascularized.

#### 3.2.6. Chordae Tendineae

The chordae tendineae and papillary muscles were first seen in 8.7 cm CVRL foetus (Figure 10), formed by modification of related trabeculae carneae. Chordae tendineae were ensheathed with a layer of endothelium and were composed of dense bundles of collagen fibers, few elastic fibers, fibroblasts, and blood vessels. These structures could not be demonstrated in all the sections due to their thin fibrous composition.

## 4. Discussion

At 0.9 cm CVRL, the buffalo foetal heart was unseptated. These findings are in agreement with the findings of human [12]. At 1.2 cm CVRL was clearly divided into common atrial chambers dorsally, primitive ventricle ventrally. Similarly [13, 14] reported that, during cardiac development from the stage of straight tube heart to early post-loop stage, there appeared zones: each one of which gave origin to specific anatomical region of definitive cardiac cavity. Another work [15] also noticed four heart segments, namely, sinus venosus, atrium, ventricle, and truncus arteriosus in caudocranial sequence. Also [16] described that continued growth forced the ventricles caudal and ventral to the atria. The truncus arteriosus was observed in foetal heart of 1.2 cm CVRL. These findings are in agreement with findings of [5, 17].

In 3.0 cm CVRL foetal heart, the endocardial cushions developed. Reference [18] reported that four pairs of endocardial cushions were found in chick heart. According to [5, 7] this phenomenon is called endocardial EMT (epithelial mesenchymal transformation). Also [12] in mouse showed that endocardial cushions acted as fundamental glue for normal septation of heart. In 7.6 cm CVRL foetus the endocardial cushions occluded the central part of the canal and separated it into two atrioventricular canals as reported by [19] in rabbit. The division of common atrium into right and left chambers via septum primum was reported in 1.9 cm CVRL buffalo foetus. The number of perforations appeared within the septum due to degeneration of cells which coalesced to form ostium secundum. Similar findings have been reported by [12] in mouse and [16] in domestic animals.

In the buffalo foetus of 3.0 cm CVRL, the ventricle was partitioned. Similarly [20] reported in mouse that ventricular septation was dependent on a single structure, interventricular septum which had both muscular and mesenchymal components. The buffalo foetus of 2.5 cm CVRL showed the truncal swellings in the lumen of truncus arteriosus. Similarly, [21] reported that the partitioning of truncus arteriosus started from the ventral aortic root towards ventricles. On contrary to this [22] reported in chicks that proximal region of pulmonary and aortic arteries did not originate from truncus arteriosus but from aortic sac.

In 8.7 cm CVRL buffalo foetus the final closure of the interventricular foramen was observed. This was in agreement with findings of [23]. However, [24] found that the final closure of interventricular foramen occurred at approximately 27 days of gestation in pig, 32 days in dog, 35 days in horse, and 45 days in human.

Cardiac jelly was also observed below the endocardium in 3.2 CVRL foetus. Also [25] reported in human embryo that at end of 3rd week of gestation the endocardium was lined by endothelial cells which rest on the cardiac jelly layer. The endothelium was found to be continued with tunica intima of pulmonary artery and aorta and endothelium of semilunar and cardiac valves in 11 cm CVRL foetus. The increased size of endocardial cells was observed at 3.2 cm CVRL foetus. The elongation of endocardial cells and presence of mitosis suggested that endocardium grew out due to increase in cell number and by redistribution of preexisting cells. The endothelial cells were elliptical to rounded shape with central nucleus and bulging towards the lumen. Similar findings have been reported by [26].

The trabeculae in the ventricles were well-differentiated at 3.2 cm CVRL buffalo foetus. The myocytes formed a dense layer which was highly trabeculated in the lumen side may be responsible for ventricular growth [26]. The myofibers were sparse in 7.6 cm and 8.7 cm CVRL buffalo foetuses due to degeneration of myocytes. Similarly, [27, 28] noticed that myocytes were sparse at 29 days of gestation in sheep. The myocardium was arranged in the compactly packed layer of muscle fibers which started from base towards the apex as reported by [29] in bovines. The myocardium of buffalo foetal heart at 13 cm CVRL showed remarkable changes. Similar findings have been reported by [28] in sheep, [30] in human. The myocardium of atria was thin as compared to ventricles and was loosely arranged in various directions, namely, circular, longitudinal, and oblique. Similar findings have been reported by [31] in rat. The nucleus of myocyte was single, large oval, or spherical with one or two nucleoli (Figure 8). This was in agreement with findings of [7] in pigs. The myocytes in right ventricle of foetal heart were larger and contained more myofibrillar material than left ventricle. Similar observations were reported in sheep by [32]. In 8.7 cm CVRL foetus the subepicardial space became enlarged which showed subepicardial thickenings at places as reported by [7] in pig. In 27 cm CVRL foetus deeper part of epicardium contained large nerve bundles, smooth muscle cells, ganglionated and nonganglionated nerve fibres, and adipose tissue. Similar findings have been reported by [33]. Epicardium of ventricles was continued with aorta and pulmonary arteries as tunica adventitia. Similar findings have been reported in pig [7].

At 19 cm CVRL, it was composed of Purkinje fibers, thin layer of myocardium, and endocardium. Similarly [34] in ox and goat reported that moderator band contained bundles of Purkinje fibers and nerve fibers separated by connective tissue. These Purkinje fibers enclosed in capsule in the right moderator band were forming the right bundle branch. Similar observations have been reported by [35] in sheep. One or two deeply stained nucleoli and mitotic figures were noticed in single nucleus which suggested that the Purkinje fibers would divide like other muscle cells and were not the nerve cells but the specialized muscle cells. This was in accordance with the findings of [7] in pigs.

At 8.7 cm CVRL, atrioventricular valves consisted of large number of mesenchymal cells with fibroblasts, collagen fibers, and elastic fibers in first group which became cellular with age. The work [36] noticed that first sign of valvular cardiogenesis was the presence of small group of cells embedded in a type II collagen positive matrix.

From 3.2 to 4.8 cm CVRL, the excavation in the atrial face of the cusps resulted in the achievement of their final shape. This excavation process was due to interaction between the endocardium of atrial face of cusps and underlying mesenchyme [37]. This was in agreement with findings of [7] in pigs. It was found that the semilunar valves were poorly vascularized.

The chordae tendineae were ensheathed with a layer of endothelium and were composed of dense bundles of collagen fibers, few elastic fibers, fibroblasts, and blood vessels. The present findings were in accordance with the findings of [38] in human and [39] in animal and human.

## Figures and Tables

**Table 1:** Details of buffalo foetuses used in study.

S. number	Group	CVRL (cm)	Age (days)
1	Group I	0.9	32
2	-do-	1.2	34
3	-do-	1.9	37
4	-do-	2.5	39
5	-do-	3.0	42
6	-do-	3.2	43
7	-do-	4.2	48
8	-do-	4.8	50
9	-do-	7.0	60
10	-do-	7.6	62
11	-do-	8.7	68
12	-do-	11.0	78
13	-do-	13.0	87
14	-do-	18.8	113
15	-do-	19.5	116
16	Group II	21.0	121
17	-do-	22.0	123
18	-do-	25.0	130
19	-do-	26.0	132
20	-do-	27.0	134
21	-do-	28.5	138
22	-do-	30.0	141
23	-do-	34.0	150
24	-do-	36.0	155
25	-do-	40.0	164
26	Group III	41.0	166
27	-do-	43.0	170
28	-do-	49.0	184
29	-do-	50.0	186
30	-do-	62	213
31	-do-	65.0	220
32	-do-	74.0	240
33	-do-	75.0	243
33	-do-	87	270
34	-do-	87.5	271
35	-do-	99.5	298

## References

[1] Taneja V. K., Birthal P. S. Role of buffalo in food security in Asia.

[2] Ahlawat P. S., Vij P. K., Tantia M. S. Protein data bank quaterly Newsletter.

[3] Nanda A. S., Nakao T. (2003). Role of buffalo in the socioeconomic development of rural Asia: current status and future prospectus. *Animal Science Journal*.

[4] Torrent-Guasp F., Kocica M. J., Corno A. F. (2005). Towards new understanding of the heart structure and function. *European Journal of Cardio-Thoracic Surgery*.

[5] Sakabe M., Matsui H., Sakata H., Ando K., Yamagishi T., Nakajima Y. (2005). Understanding heart development and congenital heart defects through developmental biology: a segmental approach. *Congenital Anomalies*.

[6] Rajpal D. K. (1990). *Pre and post hatch morpho-histogenesis of heart in fowl (Gallus domesticus) [M.S. thesis]*.

[7] Sathyamoorthy O. R. (2003). *Anatomy of the heart in pigs (Sus domesticus) [Ph.D. dissertation]*.

[8] Mandarim-de-Lacerda C. A. (1990). Human cardiac development: total volumetric increase of the heart, ventricular myocardium and endocardial cushions during the embryonic period. *Arquivos Brasileiros de Cardiologia*.

[9] Edwards M. J. (1965). Observations on the anatomy of the reproductive organs of cows: with special reference to those features sought during examination per rectum.. *New Zealand Veterinary Journal*.

[10] Soliman M. K. (1975). Studies on the physiological chemistry of the allantoic and amniotic fluids of buffalo at various periods of pregnancy. *Indian Veterinary Journal*.

[11] Luna L. G. (1968). *Manual of Histological Staining Methods of Armed Forces Institute of Pathology*.

[12] Webb S., Qayyum S. R., Anderson R. H., Lamers W. H., Richardson M. K. (2003). Septation and separation within the outflow tract of the developing heart. *Journal of Anatomy*.

[13] Christoffels V. M., Burch J. B., Moorman A. F. (2004). Architectural plan for the heart: early patterning and delineation of the chambers and the node’s. *Trends in Cardiovascular Medicine*.

[14] de la Cruz M. V., Sanchez-Gomez C., Palomino M. A. (1989). The primitive cardiac regions in the straight tube heart (Stage 9) and their anatomical expression in the mature heart: an experimental study in the chick embryo. *Journal of Anatomy*.

[15] Dyce K. M., Sack W. O., Wensing C. J. G. (1996). *Text Book of Veterinary Anatomy*.

[16] Pasquini C., Spurgeon T., Pasquini S. (2003). *Anatomy of Domestic Animals: Systemic & Regional Approach*.

[17] Brune R. M., Baldock R. A., Bard J. B. L., Davidson D. R., Kaufman M. H. (1998). Morphological appearance of the embryonic mouse heart between Theiler stages 12-14 (E 8-9) from 3-D computer reconstructions. *Journal of Anatomy*.

[18] Qayyum S. R., Webb S., Anderson R. H., Verbeek F. J., Brown N. A., Richardson M. K. (2001). Septation and valvar formation in the outflow tract of the embryonic chick heart. *Anatomical Record*.

[19] Balogh E., Sótonyi P. (2003). Histological studies on embryonic development of the rabbit heart. *Acta Veterinaria Hungarica*.

[20] Franco D., Meilhac S. M., Christoffels V. M., Kispert A., Buckingham M., Kelly R. G. (2006). Left and right ventricular contributions to the formation of the interventricular septum in the mouse heart. *Developmental Biology*.

[21] Garcia-Peláez I., Arteaga M. (1993). Experimental study of the development of the truncus arteriosus of the chick embryo heart. I. Time of appearance. *Anatomical Record*.

[22] Gómez C. S., Pliego L. P., Ramos A. C. (2005). Histological study of the proximal and distal segments of the embryonic outflow tract and great arteries. *Anatomical Record A: Discoveries in Molecular, Cellular, and Evolutionary Biology*.

[23] De La Cruz M. V., Gomez C. S., Cayre R. (1991). The developmental components of the ventricles: their significance in congenital cardiac malformations. *Cardiol Young*.

[24] Noden D. M., de Lahunta A. (1985). *The Embryology of Domestic Animals: Developmental Mechanisms and Malformations*.

[25] Carpentier A. F., Chachques J. C., Grandjean P. A., Zibaitis A., Chiu R. C. J., Kao R. L . (1997). Cardiac Bioassist. *Cellular and Molecular Cardiomyoplasty—New Approaches to Regenerate Lost Cardiac Muscles*.

[26] Icardo J. M. (1988). Heart anatomy and developmental biology. *Experientia*.

[27] Brook W. H., Connell S., Cannata J., Maloney J. E., Walker A. M. (1983). Ultrastructure of the myocardium during development from early fetal life to adult life in sheep. *Journal of Anatomy*.

[28] Burrell J. H., Boyn A. M., Kumarasamy V., Hsieh A., Head S. I., Lumbers E. R. (2003). Growth and maturation of cardiac myocytes in fetal sheep in the second half of gestation. *Anatomical Record*.

[29] Forsgren S., Thornell L. E. (1981). The development of Purkinje fibres and ordinary myocytes in the bovine fetal heart. An ultrastructural study. *Anatomy and Embryology*.

[30] Sanchez-Quintana D., Garcia-Martinez V., Climent V., Hurle J. M. (1995). Morphological changes in the normal pattern of ventricular myoarchitecture in the developing human heart. *Anatomical Record*.

[31] Knaapen M. W. M., Vrolijk B. C. M., Wenink A. C. G. (1999). Ultrastructural changes on the myocardium in the embryonic rat heart. *The Anatomical Record*.

[32] Jonker S. S., Zhang L., Louey S., Giraud G. D., Thornburg K. L., Faber J. J. (2007). Myocyte enlargement, differentiation, and proliferation kinetics in the fetal sheep heart. *Journal of Applied Physiology*.

[33] Crick S. J., Anderson R. H., Ho S. Y., Sheppard M. N. (1999). Localisation and quantitation of autonomic innervation in the porcine heart. II: endocardium, myocardium and epicardium. *Journal of Anatomy*.

[34] Jensen H., Holtet L., Hoen R. (1978). Nerve Purkinje fiber relationship in the moderator band of bovine and caprine heart. *Cell and Tissue Research*.

[35] Ansari A., Ho S. Y., Anderson R. H. (1999). Distribution of purkinje fibers in the sheep heart. *Anatomical Record*.

[36] Wessels A., Markman M. W. M., Vermeulen J. L. M., Anderson R. H., Moorman A. F. M., Lamers W. H. (1996). The development of the atrioventricular junction in the human heart. *Circulation Research*.

[37] Hurle J. M., Colvee E., Blanco A. M. (1980). Development of mouse semilunar valves. *Anatomy and Embryology*.

[38] Kawano H., Shirai T., Kawano Y., Okada R. (1996). Morphological study of vagal innervation in human semilunar valves using a histochemical method. *Japanese Circulation Journal*.

[39] Marcato P. S., Benazzi C., Bettini G. (1996). Blood and serous cysts in the atrioventricular valves of the bovine heart. *Veterinary Pathology*.

